# Central and peripheral changes in the retina and choroid in patients with diabetes mellitus without clinical diabetic retinopathy assessed by ultra-wide-field optical coherence tomography angiography

**DOI:** 10.3389/fpubh.2023.1194320

**Published:** 2023-06-13

**Authors:** Qing Zhao, Chuting Wang, Lihui Meng, Shiyu Cheng, Xingwang Gu, Youxin Chen, Xinyu Zhao

**Affiliations:** ^1^Department of Ophthalmology, Peking Union Medical College Hospital, Chinese Academy of Medical Sciences, Beijing, China; ^2^Key Laboratory of Ocular Fundus Diseases, Chinese Academy of Medical Sciences & Peking Union Medical College, Beijing, China

**Keywords:** diabetic retinopathy, ultra-wide-field, optical coherence tomography angiography, retina, choroid

## Abstract

**Background:**

To explore the central and peripheral retinal and choroidal changes in diabetic patients without clinical diabetic retinopathy (DM-NoDR) using ultra-wide-field swept-source optical coherence tomography angiography (UWF-SS-OCTA).

**Methods:**

67 DM-NoDR eyes and 32 age-matched healthy eyes were recruited. Retinal and choroidal parameters, including qualitative retinal microangiopathy, vessel flow (VFD) and linear density (VLD), thickness, and volume, were measured in the central and peripheral areas of the 24 × 20 mm^2^ UWF-SS-OCTA images.

**Results:**

DM-NoDR eyes had significantly more nonperfusion area and capillary tortuosity than controls in the central and peripheral areas (*p* < 0.05). The presence of central capillary tortuosity was associated with higher levels of serum creatinine (OR 1.049, 95%CI 1.001–1.098; *p* = 0.044) and blood urea nitrogen (OR 1.775, 95%CI 1.051–2.998; *p* = 0.032) in DM-NoDR eyes. For DM-NoDR eyes versus controls, VFD in the 300-μm annulus around the foveal avascular zone, superficial capillary plexus (SCP), and full retina, and SCP-VLD significantly decreased, while VFD in the deep capillary plexus (DCP), retinal thickness, and retinal volume increased (*p* < 0.05). Analysis in the central and peripheral areas recapitulated all these findings, except for decreased peripheral thickness and volume and no difference in peripheral DCP-VFD. In DM-NoDR eyes, choriocapillaris-VFD, choroidal thickness, and choroidal volume increased in the central area, while VFD in the large and medium choroidal vessel layer decreased in the whole image (*p* < 0.05).

**Conclusion:**

Retinal and choroidal changes already existed in the central and/or peripheral areas of DM-NoDR eyes. UWF-SS-OCTA, enabling the visualization of the peripheral fundus area, is a promising image technique for the early detection of fundus changes in DM-NoDR patients.

## 1. Introduction

Diabetes mellitus (DM) is a major global health problem, estimated to affect 642 million people worldwide by 2040 (1). As one of the most common diabetic microvascular diseases, diabetic retinopathy (DR) is the leading vision-threatening ophthalmic disease in the working-age population (2). Approximately 34.6% of diabetic patients aged over 40 years old suffer from DR, equivalent to 93 million affected people worldwide (3). Studies have found that retinal vascular changes and impaired autoregulation in DR occurred at a very early stage (4), even preceding clinically visible lesions (5). The damage caused by DR is irreversible, and the risk of progression significantly increases once DR becomes clinically observable (6). Therefore, detecting early microvascular abnormalities in DM patients without clinically visible DR (DM-NoDR) may be imperative to provide timely intervention and slow down DR progression.

Optical coherence tomography angiography (OCTA) is a novel noninvasive imaging option that can selectively visualize specific capillary plexus layers. Recently, OCTA has been widely applied in evaluating vascular pathologic changes in DM-NoDR eyes (7, 8). However, several issues remain unsolved. Firstly, ultra-wide-field (UWF) fluorescein angiography (FA) has revealed the existence of vascular abnormalities in the peripheral retina (9). However, most previous OCTA studies evaluating DM-NoDR eyes had a field of view (FOV) of only 3 × 3 mm^2^ to 12 × 12 mm^2^ (10–12). Although the montage protocol could enlarge the FOV to visualize the peripheral fundus by combining various small scans (13), it is time-consuming and cannot be widely adopted in busy routine clinical practice. Secondly, restricted by the capability of OCTA devices, previous studies mainly focused on qualitative retinal lesions and retinal vessel flow density (VFD), with few investigating the thickness and volume of different layers of the retina in DM-NoDR eyes. Thirdly, few studies explored the detailed choroidal alterations, such as VFD in the large and medium choroidal vessel layer (LMCV), choroidal vascularity volume (CVV), choroidal vascularity index (CVI), choroidal thickness and volume, needless to say, evaluating these indices by dividing the central area, peripheral area, and the whole image (14, 15). Fourthly, previous studies mainly evaluated the association between retinal changes of DM-NoDR and blood glucose-associated indices, such as fasting blood glucose (FBG) and hemoglobin A1c (HbA1c), with few exploring the relationship between fundus changes of DM-NoDR and function indices, like serum creatinine (SCr), blood urea nitrogen (BUN), etc. Finally, several authors did suggest conducting more studies to better understand the structural and hemodynamic modifications in DM-NoDR patients.

In this study, we applied novel UWF swept-source OCTA (UWF-SS-OCTA) with a FOV of 24 × 20 mm^2^ to explore central and peripheral alterations in the retina and choroid of DM-NoDR eyes. We hope our study could clarify the value of UWF-SS-OCTA for the early detection of fundus changes in DM-NoDR patients.

## 2. Methods

This cross-sectional observational study complied with the Declaration of Helsinki and has been approved by the Institutional Review Board of Peking Union Medical College Hospital (PUMCH) (K2377). All included participants provided written informed consent at the time of enrollment.

### 2.1. Participants enrollment

Patients with type 2 diabetes mellitus (T2DM), diagnosed according to the diagnostic criteria of the American Diabetes Association (16), were recruited in PUMCH in Beijing, China from June 2022 to November 2022. Only T2DM patients without clinically visible DR in the fundus photographs were included in the DM-NoDR group. During the same time, age-matched individuals without any ocular diseases (except for cataracts and non-pathologic myopia) nor systemic comorbidities comprised the healthy control group. Exclusion criteria for both groups included: (1) previous ocular surgeries that affect retinal vessels, (2) concomitant ocular diseases, such as hypertensive retinopathy, retinal arterial occlusion, age-related macular degeneration, retinal arterial macroaneurysm, previous ocular trauma, high myopia (≥6 diopters), glaucoma, uveitis, etc., (3) coexisting systemic diseases, including uncontrolled hypertension and systemic autoimmune disease, (4) inability to give informed consent or complete the full examinations, and (5) poor-quality OCTA images due to eye movements or significant ocular media opacities.

All enrolled participants underwent comprehensive ophthalmic examinations, including best-corrected visual acuity (BCVA), intraocular pressure (IOP), slit lamp fundus examination, fundus indirect ophthalmoscopy, fundus photograph, UWF-SS-OCT, and UWF-SS-OCTA. Other collected information from all DM-NoDR patients included the duration and therapy of T2DM, FBG, 2 h postprandial blood glucose (2h-PBG), HbA1c, and renal function index, including SCr, BUN, etc.

### 2.2. UWF-SS-OCT/OCTA image acquisition and analysis

**Figure 1:**
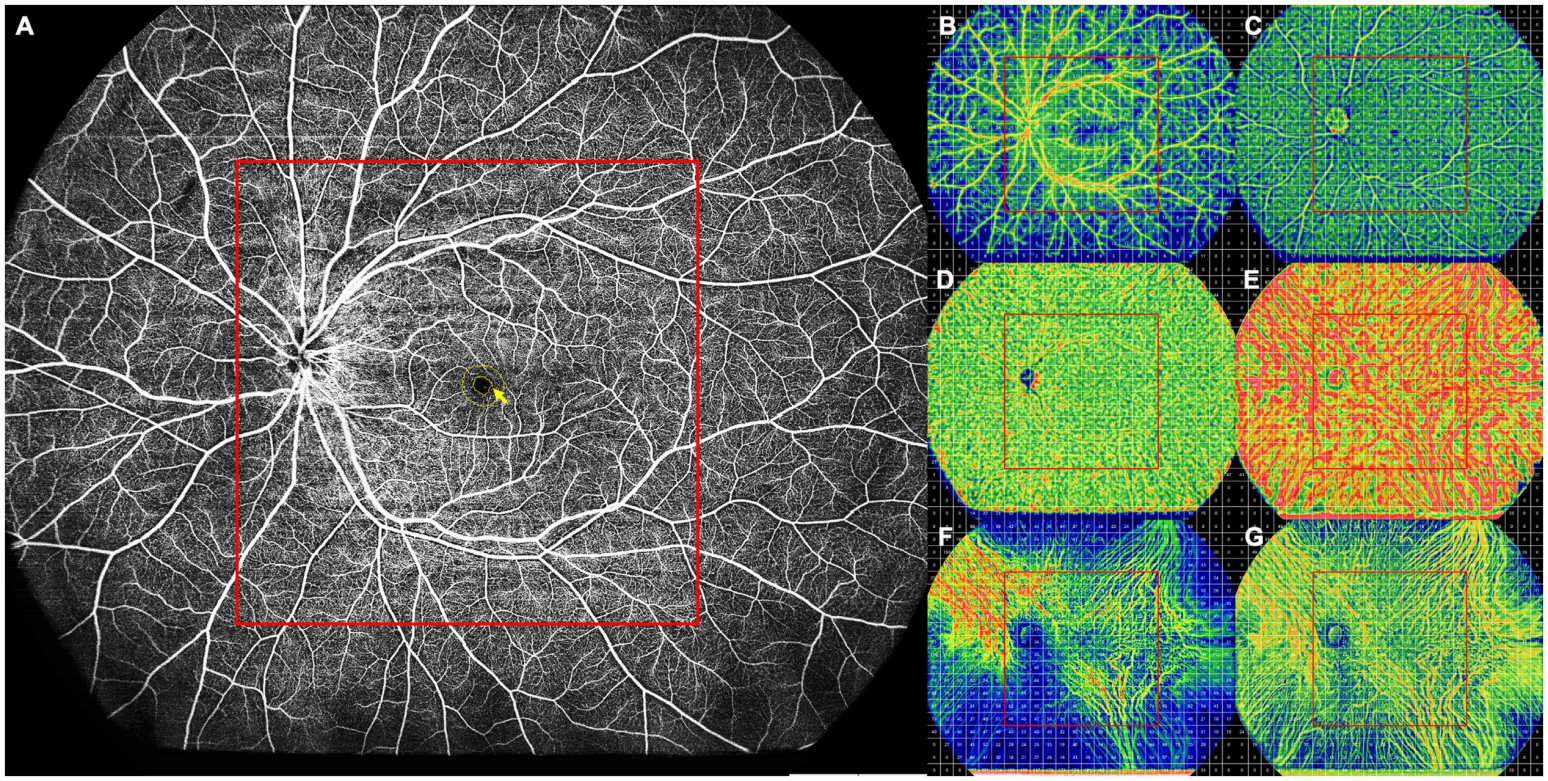
Schematic diagram of a healthy control participant. **(A)** Full retinal layer. All FAZ parameters, including the FAZ area, perimeter, AI, and FD-300, were measured on the retinal slab from the ILM to 6 μm below the outer plexiform layer. The FD-300 refers to the flow density in a 300-μm annulus around the FAZ (yellow arrow). **(B–G)** The 24 × 20 mm UWF-SS-OCTA scans of each retinal and choroidal layer were divided into 24 × 20 grids of 1 × 1 mm, and each retinal and choroidal parameter was measured in the 1 × 1 mm grid. The centered 12 × 12 grids were defined as the central area (red box), while other grids as the peripheral area. **(B)** SCP, showing VFD. **(C)** DCP, showing VFD. **(D)** CC, showing VFD. **(E)** LMCV, showing VFD. **(F)** LMCV, showing CVV. **(G)** LMCV, showing CVI. AI, acicularity index; CC, choriocapillaris; CVI, choroidal vascularity index; CVV, choroidal vascularity volume; DCP, deep capillary plexus; FAZ, foveal avascular zone; FD-300, flow density in a 300-μm annulus around the FAZ; ILM, inner limiting membrane; LMCV, large and medium choroidal vessels; SCP, superficial capillary plexus; UWF-SS-OCTA, ultra-wide-field swept-source optical coherence tomography angiography; VFD, vessel flow density.

All UWF-SS-OCT and UWF-SS-OCTA images were acquired using the BM-400 K (BMizar, TowardPi Medical Technology Co., Ltd., Beijing, China). The fovea-centered 24 × 20 mm^2^ scan pattern with a total FOV of up to 120 degrees was selected. In UWF-SS-OCTA images, the retina and choroid were automatically stratified into different sub-layers by the built-in custom segmentation of the instrument. Various retinal layers included the superficial capillary plexus layer (SCP, from the inner limiting membrane (ILM) to 9 μm below the inner plexiform layer (IPL)), deep capillary plexus layer (DCP, from 6 μm below the IPL to 9 μm below the outer plexiform layer (OPL)), and the full retina layer (from ILM to 6 μm below the OPL). The foveal avascular zone (FAZ) was automatically identified and measured using the built-in software and then any errors were corrected manually if required (see Figure 1A). The choroid was segmented into the choriocapillaris (CC) layer (between Bruch membrane (BM) and 29 μm below BM) and the LMCV layer (between 29 μm below BM and choroidoscleral interface (CSI)). Two ophthalmologists (QZ and XYZ) checked the automatic segmentation before any measurement. All acquired 24 × 20 mm^2^ UWF-SS-OCTA images were initially divided into 24 × 20 grids of 1 × 1 mm^2^, and further subdivided into central and peripheral areas. The centered 12 × 12 grids were considered as the central area, which corresponds to the FOV of the conventional 12 × 12 mm^2^ OCTA image, while the rest was defined as the peripheral area (see Figures 1B–G).

**Figure 2:**
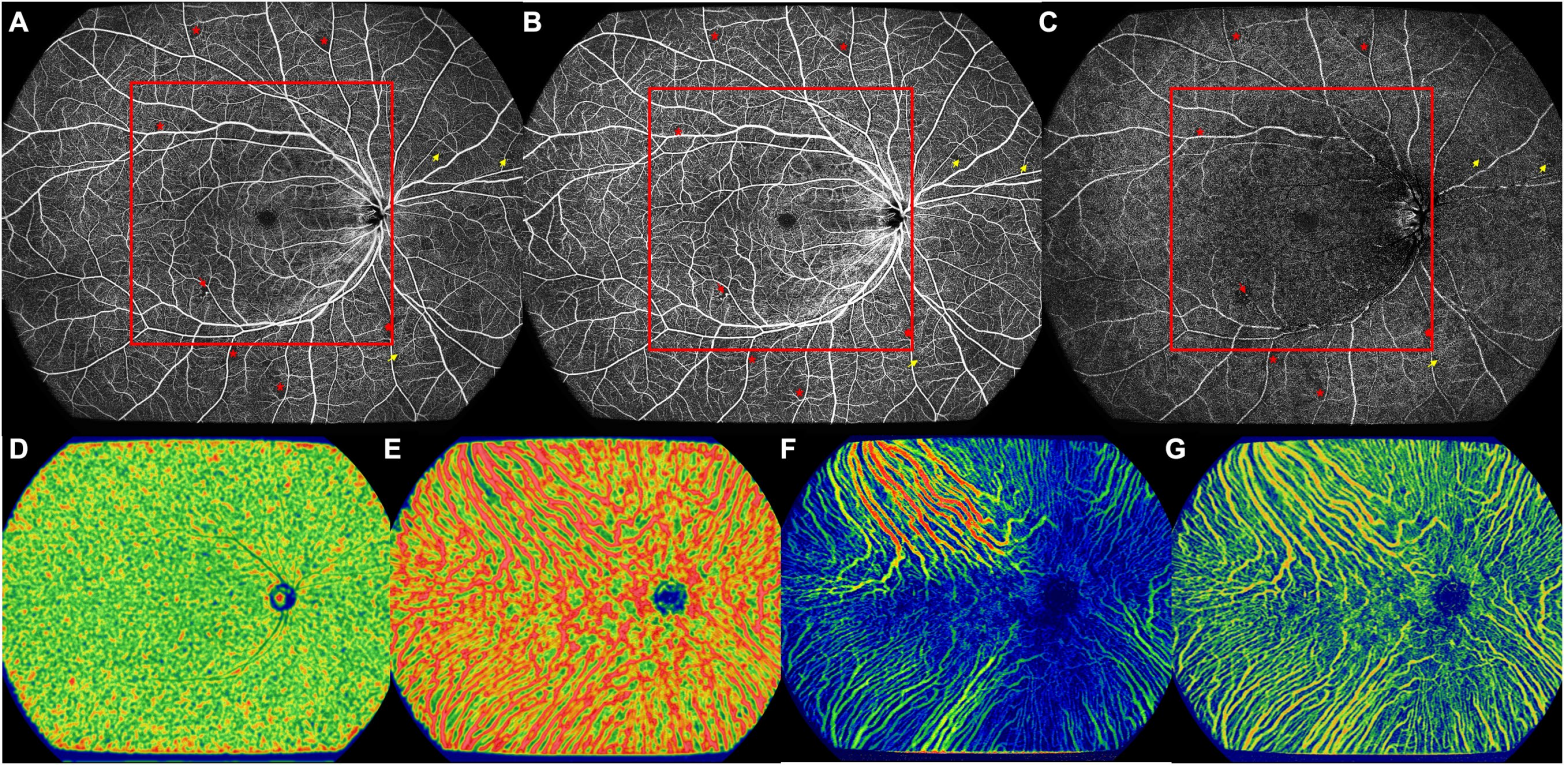
The UWF-SS-OCTA image of the right eye of a 57-year old DM-NoDR male patients with T2DM diagnosed 10 years ago. **(A)** Full retinal layer. **(B)** SCP. **(C)** DCP. Multiple retinal microvascular lesions can be noticed, including microaneurysm (red arrowhead), NPA (red asterisk), and retinal capillary tortuosity (yellow arrow). Area inside of the red box were defined as the central area, and more NPAs located in the peripheral area. **(D)** CC, showing VFD. **(E)** LMCV, showing VFD. **(F)** LMCV, showing CVV. **(G)** LMCV, showing CVI. CC, choriocapillaris; CVI, choroidal vascularity index; CVV, choroidal vascularity volume; DCP, deep capillary plexus; DM-NoDR, diabetes mellitus without clinically visible diabetic retinopathy; LMCV, large and medium choroidal vessels; NPAs, nonperfusion areas; SCP, superficial capillary plexus; T2DM, type 2 diabetes mellitus; UWF-SS-OCTA, ultra-wide-field swept-source optical coherence tomography angiography; VFD, vessel flow density.

**Figure 3:**
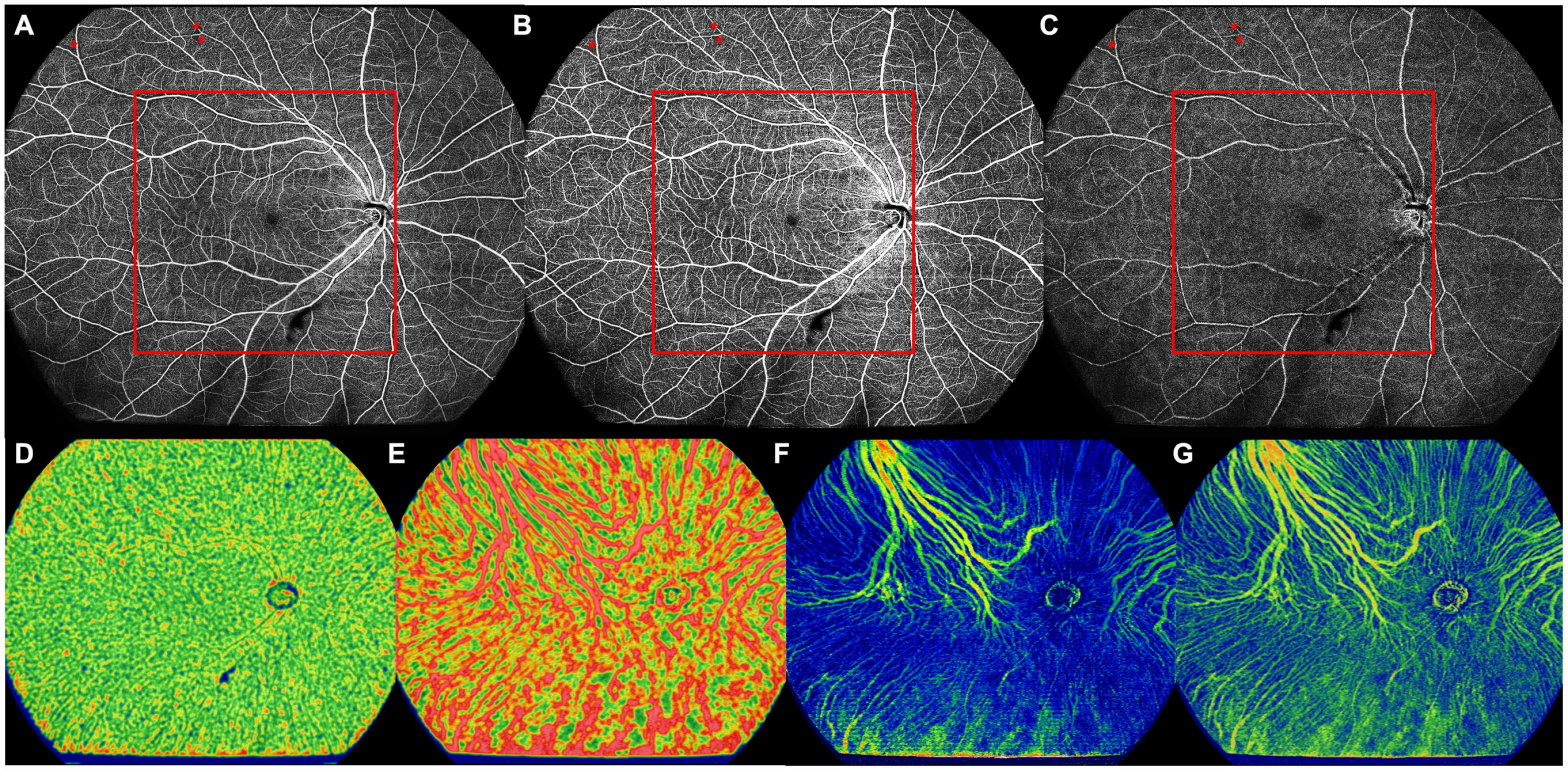
The UWF-SS-OCTA image of the right eye of a 66-year old DM-NoDR female patients with T2DM diagnosed 9 months ago. **(A)** Full retinal layer. **(B)** SCP. **(C)** DCP. NPAs (red asterisk) were noticed only in the peripheral area, but not in the central area. Area inside of the red box was defined as the central area. **(D)** CC, showing VFD. **(E)** LMCV, showing VFD. **(F)** LMCV, showing CVV. **(G)** LMCV, showing CVI. CC, choriocapillaris; CVI, choroidal vascularity index; CVV, choroidal vascularity volume; DCP, deep capillary plexus; DM-NoDR, diabetes mellitus without clinically visible diabetic retinopathy; LMCV, large and medium choroidal vessels; NPAs, nonperfusion areas; SCP, superficial capillary plexus; T2DM, type 2 diabetes mellitus; UWF-SS-OCTA, ultra-wide-field swept-source optical coherence tomography angiography; VFD, vessel flow density.

All images were evaluated and measured on the instrument display screen in a standardized and dimmed environment. Central macular thickness (CMT) and subfoveal choroidal thickness (SFCT) were measured in the UWF-SS-OCT images. Qualitative retinal parameters, including nonperfusion areas (NPAs), capillary tortuosity, and neovascularization (NV; see Figures 2, 3), were identified and classified by two masked retinal specialists (QZ and XYZ) in the UWF-SS-OCTA images. Any discrepancies were resolved by consulting a third senior retinal specialist (YXC) with over 30 years of experience in diagnosing and treating DR. FAZ-related parameters, including FAZ area, perimeter, acicularity index (AI), and the flow density in a 300-μm annulus around the FAZ (FD-300), were measured in the full retina layer. FD-300 was calculated as the ratio of the FAZ perimeter to the standard circular perimeter of the equal FAZ area. Other quantitative retinal parameters, including VFD, vessel linear density (VLD), thickness, and volume, were measured in SCP, DCP, and the full retina layer, except for VLD, which was only measured in SCP. VLD was calculated as the ratio of the length occupied by the blood vessels to the total area in the linearized vessel map (17). Choroidal parameters included the VFD in CC and LMCV layers, CVV, and CVI. CVV refers to the volume of LMCV, while the ratio of CVV to the total choroid volume is defined as CVI. Each retinal and choroidal parameter was measured in the whole OCTA image, central area, and peripheral area.

### 2.3. Data analysis

Numerical data were displayed as either mean (standard deviation, SD) or median (interquartile range, IQR), depending on the distribution. Categorical variables were presented as frequency (percentages). Snellen BCVA was converted to the respective LogMAR BCVA equivalents for statistical analysis (18). The Chi-square test or Fisher’s exact test was used to compare categorical variables. The independent-variables *t*-test or Mann–Whitney *U* test was performed to compare numerical data between DM-NoDR and healthy control groups based on the distribution. The univariable logistic regression analysis was performed to analyze the association between clinical variables and the presence of qualitative retinal microvascular lesions, presented as odds ratio (OR) with a 95% confidence interval (CI). All statistical analyses were performed using Statistical Packages for the Social Sciences (SPSS) software, version 23.0 (SPSS Inc., Chicago, IL, United States), with two-tailed *p* < 0.05 defined as statistically significant.

## 3. Results

### 3.1. Demographics

Sixty-seven eyes of 35 DM-NoDR patients and 32 eyes of 18 healthy age-matched controls were finally included. The median duration of DM in DM-NoDR patients was 8.00 years (IQR 3.50–16.00). There was no statistically significant difference in age, gender, laterality, BCVA, or IOP between these two groups. The demographics of the included participants are shown in Table 1.

**Table 1:** Demographics of the included participants.

	DM-NoDR	Control	*p*
**Patient characteristics**			
Number, n	35	18	
Age, years (mean [SD])	54.43 (13.90)	53.32 (9.67)	0.770
Gender (male), n (%)	22 (62.9%)	11 (61.1%)	1.000
DM duration, years (median [IQR])	8.00 (3.50–16.00)	NA	
DM treatment, n (%)			
*No drug therapy*	1 (2.9%)	NA	
*Oral hypoglycaemic agents*	12 (34.3%)	NA	
*Insulin*	6 (17.1%)	NA	
*Insulin and oral hypoglycaemic agents*	16 (45.7%)	NA	
Laboratory findings			
*FBG, mmol/L (mean [SD])*	7.02 (1.65)	NA	
*2h-PBG, mmol/L (mean [SD])*	10.85 (2.49)	NA	
*HbA1c, % (median [IQR])*	7.50 (6.30–8.50)	NA	
*SCr, umol/L (median [IQR])*	66.00 (57.00–73.00)	NA	
*BUN, mg/L (mean [SD])*	5.48 (1.94)	NA	
*UMAlb, mg/L (median [IQR])*	4.25 (1.90–14.40)	NA	
*UCr, mmol/L (mean [SD])*	8.92 (4.68)	NA	
*ACR, mg/g Cr (median [IQR])*	3.50 (2.00–20.00)	NA	
*24hUP, g/24 h (median [IQR])*	0.13 (0.09–0.20)	NA	
**Eye Characteristics**			
Number, *n*	67	32	
Laterality (OD), *n* (%)	35 (52.2%)	17 (53.1%)	1.000
BCVA (median[IQR])	0.00 (0.00–0.10)	0.00 (0.00–0.16)	0.922
IOP (median[IQR])	13.40 (11.80–15.80)	13.90 (12.00–16.00)	0.411

2h-PBG, 2 h postprandial blood glucose; 24hUP, 24 h urine protein; ACR, albumin creatinine ratio; BCVA, best-corrective visual acuity; BUN, blood urea nitrogen; DM, diabetic mellitus; DM-NoDR, diabetes mellitus without clinically visible diabetic retinopathy; FBG, fasting blood glucose; IOP, intraocular pressure; IQR, interquartile range; NA, not applicable; SCr, serum creatinine; SD, standard deviation; UCr, urine creatinine; UMAlb, urine microalbumin.

### 3.2. Qualitative retinal parameters

A significantly increased number of NPA and capillary tortuosity was noticed in DM-NoDR eyes in the whole, central, and peripheral areas compared to healthy control eyes (*p* < 0.05; see Table 2). NV was not found in DM-NoDR eyes or controls. In DM-NoDR eyes, 38 (56.7%) had peripheral capillary tortuosity, which was significantly higher than the incidence of central capillary tortuosity (13, 19.4%) (*p* = 0.030). NPAs and NV were distributed equally in the central and peripheral areas in DM-NoDR eyes (*p* > 0.05). Microaneurysms (MAs) were found in 5 (7.5%) of the DM-NoDR eyes, with no difference in the central or peripheral area (3(4.5%) vs. 3(4.5%); *p* = 0.130).

**Table 2:** Retinal parameters of the diabetic eyes without clinical diabetic retinopathy and control eyes.

	DM-NoDR eyes	Control eyes	*p*
**Qualitative retinal parameters**			
NPA, *n* (%)			
*Whole image*	60 (89.6%)	4 (12.5%)	< 0.001^a^
*Central area*	35 (52.2%)	0 (0.0%)	< 0.001^a^
*Peripheral area*	58 (86.6%)	4 (12.5%)	< 0.001^a^
Capillary tortuosity, *n* (%)		
*Whole image*	40 (59.7%)	0 (0.0%)	< 0.001^a^
*Central area*	13 (19.4%)	0 (0.0%)	0.008^a^
*Peripheral area*	38 (56.7%)	0 (0.0%)	< 0.001^a^
NV, *n* (%)
*Whole image*	0 (0.0%)	0 (0.0%)	NA
*Central area*	0 (0.0%)	0 (0.0%)	NA
*Peripheral area*	0 (0.0%)	0 (0.0%)	NA
**Quantitative retinal parameters**			
FAZ			
*Area, mm^2^ (mean [SD])*	0.34 (0.12)	0.32 (0.13)	0.504
*Perimeter, mm (median [IQR])*	2.46 (2.15–2.73)	2.42 (2.10–2.78)	0.563
*AI (median[IQR])*	1.18 (1.15–1.26)	1.19 (1.15–1.27)	0.746
*FD-300, % (median [IQR])*	11.57 (8.29–16.33)	30.88 (28.26–34.75)	< 0.001^a^
**Vessel flow density, %**			
SCP			
*Whole image (median [IQR])*	25.63 (24.34–26.91)	34.44 (33.40–36.02)	< 0.001^a^
*Central area (median [IQR])*	34.27 (32.14–35.61)	40.08 (38.45–41.91)	< 0.001^a^
*Peripheral area (median [IQR])*	21.95 (20.88–23.18)	31.64 (29.72–33.05)	< 0.001^a^
DCP			
*Whole image (median [IQR])*	25.77 (24.00–27.21)	21.89 (20.11–24.68)	< 0.001^a^
*Central area (median [IQR])*	33.22 (30.17–34.60)	22.42 (20.09–23.98)	< 0.001^a^
*Peripheral area (median [IQR])*	22.83 (21.06–24.30)	21.80 (19.84–24.42)	0.148
Retina			
*Whole image (median [IQR])*	26.05 (24.01–27.21)	34.94 (33.19–35.94)	< 0.001^a^
*Central area (median [IQR])*	33.71 (31.38–35.03)	40.07 (37.64–41.16)	< 0.001^a^
*Peripheral area (median [IQR])*	22.52 (29.82–33.51)	32.52 (29.82–33.51)	< 0.001^a^
**Vessel linear density, %**			
SCP			
*Whole image (median [IQR])*	6.04 (5.75–6.35)	8.77 (8.37–9.02)	< 0.001^a^
*Central area (median [IQR])*	8.15 (7.75–8.38)	10.03 (9.65–10.33)	< 0.001^a^
*Peripheral area (median [IQR])*	5.13 (4.89–5.49)	8.08 (7.64–8.36)	< 0.001^a^
Thickness, μm			
*CMT, μm (median [IQR])*	239.00 (228.00–254.00)	230.50 (215.00–243.25)	0.015^a^
SCP			
*Whole image (mean [SD])*	68.67 (3.90)	65.56 (3.99)	< 0.001^a^
*Central area (mean [SD])*	97.92 (6.47)	65.01 (8.43)	< 0.001^a^
*Peripheral area (mean [SD])*	56.14 (3.68)	65.79 (4.74)	< 0.001^a^
DCP			
*Whole image (median [IQR])*	159.89 (155.72–165.91)	155.75 (151.37–159.09)	0.003^a^
*Central area (median [IQR])*	177.55 (173.27–184.36)	155.45 (151.50–159.06)	< 0.001^a^
*Peripheral area (median [IQR])*	152.32 (148.35–157.69)	154.51 (148.35–157.69)	0.044^a^
Retina			
*Whole image (median [IQR])*	229.88 (224.02–233.60)	222.93 (215.88–227.74)	< 0.001^a^
*Central area (mean [SD])*	276.48 (9.70)	220.67 (10.67)	< 0.001^a^
*Peripheral area (median [IQR])*	208.21 (203.33–212.63)	220.33 (216.44–230.33)	0.008^a^
**Volume, mm** **3**			
SCP			
*Whole image (mean [SD])*	0.068 (0.004)	0.065 (0.004)	0.001^a^
*Central area (mean [SD])*	0.098 (0.006)	0.065 (0.008)	< 0.001^a^
*Peripheral area (mean [SD])*	0.055 (0.004)	0.065 (0.005)	< 0.001^a^
DCP			
*Whole image (median [IQR])*	0.16 (0.15–0.16)	0.15 (0.15–0.16)	0.008^a^
*Central area (median [IQR])*	0.18 (0.17–0.18)	0.16 (0.15–0.16)	< 0.001^a^
*Peripheral area (median [IQR])*	0.15 (0.14–0.15)	0.15 (0.15–0.16)	0.007^a^
Retina			
*Whole image (median [IQR])*	0.22 (0.22–0.23)	0.22 (0.21–0.22)	0.003^a^
*Central area (mean [SD])*	0.28 (0.01)	0.22 (0.01)	< 0.001^a^
*Peripheral area (median [IQR])*	0.20 (0.20–0.21)	0.21 (0.21–0.22)	< 0.001^a^

a *p* < 0.05.

AI, acicularity index; CMT, central macular thickness; DCP, deep capillary plexus; DM-NoDR, diabetes mellitus without clinically visible diabetic retinopathy; FAZ, foveal avascular zone; FD-300, flow density in a 300-μm annulus around the FAZ; IQR, interquartile range; NA, not available; NPA, nonperfusion area; NV, neovascularization; SCP, superficial capillary plexus; SD, standard deviation.

In the univariable logistic regression analysis, higher levels of SCr (OR 1.049, 95%CI 1.001–1.098; *p* = 0.044) and BUN (OR 1.775, 95%CI 1.051–2.998; *p* = 0.032) were significantly associated with the presence of central capillary tortuosity. No significant association existed between age, gender, duration of DM, DM treatment, or other laboratory findings and the appearance of retinal microvascular lesions (*p* > 0.05; see Supplementary Tables S1–S3).

### 3.3. Quantitative retinal parameters

The median FD-300 in DM-NoDR eyes was 11.57 (IQR 8.29–16.33), significantly lower than that in the control eyes (median 30.88, IQR 28.26–34.75; *p* < 0.001). There was no significant difference in the FAZ area, perimeter, and AI between these two groups (*p* > 0.05).

Compared with the control group, SCP-VFD, SCP-VLD, and full-retinal VFD significantly decreased, while DCP-VFD significantly increased in the DM-NoDR group in the whole UWF-SS-OCTA images (*p* < 0.001). Further comparison in central and peripheral areas recapitulated all these findings, except for no significant difference in peripheral DCP-VFD (*p* > 0.05).

The DM-NoDR eyes had a median CMT of 239.00 (IQR 228.00–254.00) μm, which was significantly thicker than the control eyes (median 230.50, IQR 215.00–243.25; *p* = 0.015). Compared to controls, the thickness and volume of all retinal layers increased in both the whole and central areas, while decreasing in the peripheral area in DM-NoDR eyes (*p* < 0.05; see Table 2).

### 3.4. Choroidal parameters

The CC-VFD, choroidal thickness, and choroidal volume were significantly higher in the central area of DM-NoDR eyes than in controls (*p* < 0.05). However, no significant difference was found in the whole image and peripheral area (*p* > 0.05). Besides, the DM-NoDR group had a significantly lower LMCV-VFD than controls in the whole image (mean 62.14, SD 2.60 vs. mean 63.37, SD 3.11; *p* = 0.042). No significant difference existed in SFCT, CVV, and CVI between these two groups (*p* > 0.05; see Table 3).

**Table 3:** Choroidal parameters of the diabetic eyes without clinical diabetic retinopathy and control eyes.

	DM-NoDR eyes	Control eyes	*p*
SFCT, μm (mean [SD])	262.45 (89.16)	234.22 (78.16)	0.129
**Vessel flow density, %**			
Choriocapillaris layer			
*Whole image (mean [SD])*	43.45 (1.02)	43.60 (1.12)	0.506
*Central area (mean [SD])*	46.29 (0.68)	45.45 (0.79)	< 0.001^a^
*Peripheral area (mean [SD])*	42.22 (1.26)	42.67 (1.51)	0.128
Large and medium choroidal vessel layer			
*Whole image (mean [SD])*	62.14 (2.60)	63.37 (3.11)	0.042^a^
*Central area (median [IQR])*	68.25 (66.06–69.53)	67.71 (66.35–69.77)	0.793
*Peripheral area (median [IQR])*	60.07 (58.40–61.64)	61.02 (58.86–64.02)	0.061
CVV, mm^3^			
*Whole image (median [IQR])*	67.06 (59.01–77.41)	67.06 (59.01–77.41)	0.198
*Central area (mean [SD])*	80.99 (24.37)	79.94 (18.36)	0.831
*Peripheral area (median [IQR])*	58.87 (50.80–67.26)	63.37 (55.49–72.78)	0.060
CVI, %			
*Whole image (mean [SD])*	28.56 (3.87)	30.12 (4.13)	0.070
*Central area (mean [SD])*	31.81 (4.32)	32.87 (4.71)	0.270
*Peripheral area (median [IQR])*	28.63 (25.09–31.05)	26.96 (24.95–28.71)	0.057
Choroidal thickness, μm			
*Whole image (mean [SD])*	186.24 (33.08)	173.87 (27.71)	0.070
*Central area (median [IQR])*	215.35 (172.19–232.75)	191.20 (161.83–206.54)	0.020^a^
*Peripheral area (mean [SD])*	176.35 (29.13)	168.22 (28.79)	0.196
Choroidal volume, mm^3^			
*Whole image (mean [SD])*	0.18 (0.03)	0.17 (0.03)	0.093
*Central area (mean [SD])*	0.21 (0.05)	0.19 (0.03)	0.020^a^
*Peripheral area (mean [SD])*	0.17 (0.03)	0.16 (0.03)	0.298

a *p* < 0.05.

CVI, choroidal vascularity index; CVV, choroidal vascularity volume; DM-NoDR, diabetes mellitus without clinically visible diabetic retinopathy; IQR, interquartile range; SD, standard deviation; SFCT, subfoveal choroidal thickness.

## 4. Discussion

This is the first study, to the best of our knowledge, to utilize UWF-SS-OCTA with a FOV of 24 × 20 mm^2^ to analyze early central and peripheral alterations in the retina and choroid of DM-NoDR patients. We found that microvascular impairments, including NPA and capillary tortuosity, had already begun in DM-NoDR eyes, both in the central and peripheral areas. Higher levels of SCr and BUN were associated with the presence of central capillary tortuosity in DM-NoDR eyes. Additionally, quantitative retinal alterations were observed in DM-NoDR eyes: FD-300, SCP-VFD, SCP-VLD, and full-retinal VFD significantly decreased, while DCP-VFD, CMT, retinal thickness, and retinal volume increased. Analysis of the central and peripheral areas recapitulated all these findings, except for decreased peripheral thickness and volume and no difference in peripheral DCP-VFD. Regarding choroidal changes in DM-NoDR eyes, CC-VFD, choroidal thickness, and choroidal volume increased in the central area, while LMCV-VFD decreased in the whole image.

Currently, ophthalmoscopically visible retinal MAs are recognized as the first microvascular sign of DR. Our study found that microvascular abnormalities, including NPA and capillary tortuosity, already began in DM-NoDR eyes, consistent with previous studies (19, 20). Furthermore, we even identified 5 eyes with MAs in UWF-SS-OCTA but not in fundus examination in the DM-NoDR group, which might suggest the superiority of OCTA to fundus examination for detecting retinal microvascular alterations. Furthermore, according to current diagnostic criteria, these eyes may still be categorized as NoDR. However, these findings may challenge the current DR classification system and suggest the need for a new DR classification consensus based on OCTA. With the assistance of UWF-SS-OCTA, which enables visualization of the peripheral fundus area, we further found that capillary tortuosity was more prevalent in the peripheral area than in the central area in DM-NoDR eyes. Zhuang et al. (21) further found that diabetic eyes with peripheral lesions had worse retinal function, such as delayed implicit time. Therefore, a narrow FOV might lead to the misidentification of peripheral retinal microvascular alterations and ultimately the misclassification of DR severity (22).

Chronic hyperglycemia can cause microvascular changes in both the glomerulus and retina (23). In the present study, higher levels of SCr and BUN, indicators of renal dysfunction, were associated with the presence of central capillary tortuosity in DM-NoDR eyes. Similarly, Tan et al. (24) also demonstrated that DM-NoDR patients with higher levels of BUN had more severe capillary abnormalities. Furthermore, increases in SCr and BUN were also found to be associated with increased DR severity (25). These findings provide a mutual corroboration between diabetic nephropathy and DR. HbA1c is a well-known and validated biomarker for the incidence and progression of DR. Cheung et al. (26) previously reported that a 1% decrease in HbA1c may be equivalent to a 40% decrease in DR risk in diabetic patients. However, in DM-NoDR eyes, our study found no significant association between HbA1c and the presence of retinal microvascular changes in our study, consistent with previous studies (27). The association of systemic conditions and retinal microvascular impariments needs further validation in prospective studies.

FAZ, which refers to the macular capillary-free zone surrounded by interconnected capillaries, reflects the retinal microcirculation and has been shown to enlarge with DR aggravation (28). However, whether the FAZ is altered in DM-NoDR eyes remains controversial. Some studies have reported that the FAZ area was enlarged in DM-NoDR eyes compared to controls (10, 29, 30), while others have found no significant change (31–33). In our study, we observed no significant differences in the area, perimeter, or AI of the FAZ between DM-NoDR eyes and controls. We hypothesized that the inconsistencies in previous studies might be due to the high variability of FAZ area in healthy individuals (34). In addition, our study showed that the FD-300 was lower in DM-NoDR eyes than in controls, which is consistent with the results of a previous meta-analysis (35). These findings suggest that reduced foveal VFD might be one of the earliest sensitive indicators of early retinal microvasculature abnormalities in diabetic patients before the development of clinically visible DR.

In the present study, we found that both SCP-VFD and full-retinal VFD were significantly reduced in the central 12 × 12 mm^2^ retinal area of DM-NoDR eyes compared to controls, similar to previous studies using OCTA with a FOV of 3 × 3 mm^2^ or 6 × 6 mm^2^ (20, 24, 27, 36). Using UWF-SS-OCTA, we further observed that DM-NoDR eyes also had reduced SCP-VFD and full-retinal VFD in the peripheral area as well. The retinal blood flow reduction might be attributed to the disruption of retinal neurovascular autoregulation, capillary nonperfusion, capillary dropout, or vasoconstriction by various mechanism (37, 38). Chu et al. (17) previously reported that VLD might be a more sensitive parameter to measure capillary perfusion changes. In our study, DM-NoDR eyes had significantly reduced SCP-VLD, consistent with the appearance of nearly 90% NPA. However, Dai et al. (39) found similar SCP-VLD in DM-NoDR eyes compared with controls using the 6 × 6 mm^2^ OCTA scanning protocol. This inconsistency might be attributed to the longer duration of DM in our study participants (median 8.00 years, IQR 3.50–16.00 vs. mean 2.1 years, SD 1.2).

Several previous studies have reported unchanged or lower parafoveal DCP-VFD in DM-NoDR eyes (15, 36, 40). However, we found significantly increased central and whole DCP-VFD in DM-NoDR eyes, in accordance with previous studies (20). The increased DCP-VFD, possibly through the dilation of existing capillaries (41), might be a compensatory mechanism that enables DM-NoDR eyes to meet the metabolic needs of the retina in the setting of decreased SCP-VFD. However, increased blood flow through the dilated retinal capillaries in the DCP, when reaching its compensatory limit, could damage the integrity of microvascular walls and finally lead to the appearance of NPAs, MAs, and dot-and-blot hemorrhages (11). At a more advanced stage of DR, reduced DCP-VFD would decrease the re-absorption of intraretinal fluid in DCP and lead to the formation of intraretinal edema (42).

In our study, the retinal thickness and volume of DM-NoDR eyes significantly increased in the central 12 × 12 mm^2^ area, while decreasing significantly in the peripheral area. Previous studies have reported that chronic hyperglycemia-induced chronic inflammation, oxidative stress, and ischemia can cause neural apoptosis and glial cell activation, leading to thinning of the inner retinal layer (43, 44). Our findings might suggest that this hyperglycemia-induced neural apoptosis may originate from the periphery.

The choroid is an important vascular tissue that supplies blood to the outer retina, and the increased choriocapillaris flow deficit has been widely reported in DR (45, 46). However, the previously reported changes in CC-VFD in DM-NoDR eyes were contradictory (35, 39, 47, 48), which might be explained by the different measured hemodynamic parameters. Histopathologic studies showed that postmortem diabetic subjects, even without DR, were associated with more pronounced CC dropouts than nondiabetic subjects (49). Meanwhile, postmortem diabetic subjects also had more beaded and tortuous CC. Therefore, the alteration of CC-VFD might depend on the balance between CC dropouts and beaded and tortuous CC. In our study, we found a significantly higher CC-VFD in DM-NoDR eyes in the central 12 × 12 mm^2^ area of the UWF-SS-OCTA image. Besides, we also found significantly decreased LMCV-VFD in the whole image, resulting from the decrease in peripheral LMCV-VFD, although without statistical difference. Further studies are needed to explore the changes in CC-VFD and LMCV-VFD in DM-NoDR eyes.

A recent systematic review and meta-analysis reviewed that diabetic patients diagnosed with DR had thicker choroids than those without DR (50). Besides, the choroidal thickness significantly increased as DR severity worsened (51). Our study further reported that DM-NoDR eyes also had increased choroidal thickness and volume than controls in the central 12 × 12 mm^2^ area, in accordance with the finding of Xu et al. (52). The increased choroidal thickness and volume with unchanged LMCV-VFD and CVV in the central area imply that the increase is mainly in the stromal component. However, the underlying mechanism and clinical importance of this alteration remain unknown, and further investigations are warranted.

We acknowledge several limitations in our study. Firstly, as a hospital-based study, the healthy controls we enrolled may not perfectly represent normal individuals. Secondly, non-capillary large vessels were not removed in the analysis of retinal VFD. Considering the different anatomical structures of large vessels and capillaries, their structural and hemodynamic alterations from hyperglycemia may not exactly be the same. And therefore, the blood flow in large retinal vessels might influence the analysis of retinal microvasculature in DM-NoDR eyes. Thirdly, the automated built-in custom segmentation of the instrument only stratified the retina into SCP and DCP, while the middle capillary plexus (MCP) has recently been introduced (53) and found to be quantitatively and qualitatively different from SCP and DCP. Future studies are warranted to further assess the MCP alterations. Finally, the longer image acquisition time and larger FOV of UWF-SS-OCTA images might result in more artifacts, especially in the periphery. In this study, we excluded areas with obvious artifacts in the measurement of quantitative retinal and choroidal parameters to ensure reliable interpretation. Future technical advancements, like novel self-navigation, are needed to reduce these artifacts and enable the UWF-SS-OCTA to become a mass screening tool for DR.

In conclusion, retinal and choroidal changes already exist in the central and/or peripheral fundus area of DM-NoDR eyes. The apperance of central retinal capillary tortuosity was associated with higher levels of SCr and BUN. UWF-SS-OCTA, which enables the visualization of the peripheral fundus area, presents a promising imaging option to characterize structural and hemodynamic changes in DM-NoDR patients.

## Data availability statement

The original contributions presented in the study are included in the article/Supplementary material, further inquiries can be directed to the corresponding authors.

## Ethics statement

The studies involving human participants were reviewed and approved by The Institutional Review Board of Peking Union Medical College Hospital (PUMCH), Approval number: K2377. The patients/participants provided their written informed consent to participate in this study.

## Author contributions

QZ, XZ, and YC designed the study. QZ, CW, LM, SC, and XG collected data. QZ performed the statistical analysis and drafted the manuscript. All authors contributed to the article and approved the submitted version.

## Funding

This study was supported by the Fundamental Research Funds for the Central Universities (grant no. 3332022008) and the National College Students Innovation and Entrepreneurship Training Program (grant no. 2022zglc06028).

## Conflict of interest

The authors declare that the research was conducted in the absence of any commercial or financial relationships that could be construed as a potential conflict of interest.

## Publisher’s note

All claims expressed in this article are solely those of the authors and do not necessarily represent those of their affiliated organizations, or those of the publisher, the editors and the reviewers. Any product that may be evaluated in this article, or claim that may be made by its manufacturer, is not guaranteed or endorsed by the publisher.

## Supplementary material

The Supplementary material for this article can be found online at: https://www.frontiersin.org/articles/10.3389/fpubh.2023.1194320/full#supplementary-material

## Figures and Tables

**Table:** 

AI	acicularity index
BCVA	best-corrected visual acuity
BM	Bruch membrane
BUN	blood urea nitrogen
CC	choriocapillaris
CI	confidence interval
CMT	central macular thickness
CSI	choroidoscleral interface
CVI	choroidal vascularity index
CVV	choroidal vascularity volume
DCP	deep capillary plexus
DM	diabetes mellitus
DM-NoDR	diabetes mellitus without clinically visible diabetic retinopathy
DR	diabetic retinopathy
FA	fluorescein angiography
FAZ	foveal avascular zone
FBG	fasting blood glucose
FD-300	flow density in a 300-μm annulus around the FAZ
FOV	field of view
HbA1c	hemoglobin A1c
ILM	inner limiting membrane
IOP	intraocular pressure
IPL	inner plexiform layer
IQR	interquartile range
LMCV	large and medium choroidal vessels
MAs	microaneurysms
MCP	middle capillary plexus
NPA	nonperfusion area
NV	neovascularization
OCTA	optical coherence tomography angiography
OPL	outer plexiform layer
OR	odds ratio
SCP	superficial capillary plexus
SCr	serum creatinine
SD	standard deviation
SFCT	subfoveal choroidal thickness
T2DM	type 2 diabetes mellitus
UWF	ultra-wide-field
UWF-SS-OCTA	ultra-wide-field swept-source optical coherence tomography angiography
VFD	vessel flow density
VLD	vessel linear density
2h-PBG	2 h postprandial blood glucose
