# Supplementary material for: Central and peripheral changes in the retina and choroid in patients with diabetes mellitus without clinical diabetic retinopathy assessed by ultra-wide-field optical coherence tomography angiography
Source: Front Public Health. 2023 Jun 13;11:1194320. doi: 10.3389/fpubh.2023.1194320 (PMC10293646; doi:10.3389/fpubh.2023.1194320)
Supplement: Supplementary file 1 [file Data_Sheet_1.PDF]

*Supplementary Material*

# Central and Peripheral Changes in the Retina and Choroid in Patients with Diabetes Mellitus without Clinical Diabetic Retinopathy Assessed by Ultra-Wide-Field Optical Coherence Tomography Angiography

**Qing Zhao[1,2], Chuting Wang[1,2], Lihui Meng[1,2], Shiyu Cheng[1,2], Xingwang Gu[1,2], Youxin Chen[1,2,*,†], Xinyu Zhao[1,2,*,†]**

*** Correspondence:**
Xinyu Zhao[*], Youxin Chen[*]
zhaoxinyu@pumch.cn, chenyx@pumch.cn

# 1 Supplementary Tables

## 1.1 Supplementary Table 1. Univariable logistic regression analysis of potential related factors in DM-NoDR eyes with and without retinal microvascular changes in the whole image.

| | NPA | No-NPA | OR | *P* | Capillary tortuosity | No-Capillary tortuosity | OR | P |
|---|---|---|---|---|---|---|---|---|
| **Number, n** | 30 | 5 | | | 23 | 12 | | |
| **Age, years (mean[SD])** | 54.30 (14.18) | 55.20 (13.52) | 1.005 (0.936-1.079) | 0.892 | 56.26 (12.45) | 50.92 (16.32) | 1.028 (0.977-1.083) | 0.286 |
| **Gender (male), n (%)** | 19 (63.3%) | 3 (60.0%) | 1.152 (0.166-7.990) | 0.886 | 16 (69.6%) | 6 (50.0%) | 2.286 (0.542-9.633) | 0.260 |
| **DM duration, years (median[IQR])** | 9.00 (2.50-16.00) | 7.33 (3.50-13.50) | 1.028 (0.909-1.162) | 0.662 | 10.00 (1.00-17.00) | 7.17 (4.25-10.75) | 1.041 (0.951-1.140) | 0.379 |
| **DM treatment, n(%)** | | | | | | | | |
| *No drug therapy* | 1 (3.3%) | 0 (0.0%) | NA | 1.000 | 1 (4.3%) | 0 (0.0%) | NA | 1.000 |
| *Oral hypoglycaemic agents* | 10 (33.3%) | 2 (40.0%) | 0.750 (0.107-5.238) | 0.772 | 7 (30.4%) | 5 (41.7%) | 0.613 (0.144-2.614) | 0.508 |
| *Insulin* | 6 (20.0%) | 0 (0.0%) | NA | 0.561 | 4 (17.4%) | 2 (16.7%) | 1.053 (0.164-6.776) | 0.957 |
| *Insulin and oral hypoglycaemic agents* | 13 (43.3%) | 3 (60.0%) | 0.510 (0.074-3.510) | 0.494 | 11 (47.8%) | 5 (41.7%) | 1.283 (0.314-5.253) | 0.729 |
| **Laboratory findings** | | | | | | | | |
| *FBG, mmol/L (mean[SD])* | 6.86 (1.71) | 8.02 (0.63) | 0.632 (0.337-1.188) | 0.154 | 6.77 (1.74) | 7.52 (1.39) | 0.746 (0.474-1.173) | 0.204 |
| *2h-PBG, mmol/L (mean[SD])* | 10.90 (2.43) | 10.54 (3.12) | 1.065 (0.713-1.591) | 0.760 | 11.19 (2.62) | 10.21 (2.18) | 1.192 (0.871-1.631) | 0.272 |
| *HbA1c, % (median[IQR])* | 7.50 (6.25-8.50) | 7.50 (6.75-9.85) | 0.853 (0.505-1.441) | 0.552 | 7.45 (6.30-8.28) | 7.75 (6.13-10.40) | 0.709 (0.460-1.095) | 0.121 |
| *SCr, umol/L (median[IQR])* | 67.00 (57.00-73.50) | 62.00 (37.50-86.50) | 1.018 (0.964-1.075) | 0.514 | 69.00 (57.00-76.00) | 62.00 (49.25-71.00) | 1.030 (0.986-1.075) | 0.186 |
| *BUN, mg/L (mean[SD])* | 5.39 (1.99) | 6.02 (1.69) | 0.852 (0.534-1.358) | 0.500 | 5.65 (2.10) | 5.15 (1.63) | 1.156 (0.783-1.706) | 0.466 |
| *UMAlb, mg/L (median[IQR])* | 3.90 (1.90-13.90) | 4.70 (3.00-12.35) | 0.987 (0.916-1.064) | 0.738 | 4.25 (2.20-16.65) | 4.15 (0.93-16.93) | 1.007 (0.992-1.022) | 0.383 |
| *UCr, mmol/L (mean[SD])* | 9.28 (4.55) | 5.95 (5.68) | 1.250 (0.854-1.830) | 0.251 | 10.04 (4.38) | 6.90 (4.74) | 1.204 (0.963-1.505) | 0.103 |
| *ACR, mg/g Cr (median[IQR])* | 4.00 (2.00-20.00) | 3.00 (3.00-84.00) | 1.002 (0.993-1.011) | 0.69 | 4.50 (2.00-20.00) | 3.00 (1.75-59.25) | 1.013 (0.986-1.039) | 0.351 |
| *24hUP, g/24h (median[IQR])* | 0.13 (0.08-0.21) | NA | NA | NA | 0.11 (0.08-0.19) | 0.71 (0.14-1.27) | 0.001 (0.000-1.030×$10^6$) | 0.525 |

**Abbreviations:** 2h-PBG: 2-hour postprandial blood glucose; 24hUP: 24 hours urine protein; ACR: albumin creatinine ratio; BUN: blood urea nitrogen; DM: diabetic mellitus; DM-NoDR: diabetes mellitus without clinically visible diabetic retinopathy; FBG: fasting blood glucose; IQR: interquartile range; NA: not applicable; NPA: nonperfusion area; OR: odds ratio; SCr: serum creatinine; SD: standard deviation; UCr: urine creatinine; UMAlb: urine microalbumin.

## 1.2 Supplementary Table 2. Univariable logistic regression analysis of potential related factors in DM-NoDR eyes with and without retinal microvascular changes in the central area.

| | NPA | No-NPA | OR | *P* | Capillary tortuosity | No-Capillary tortuosity | OR | *P* |
|---|---|---|---|---|---|---|---|---|
| **Number, n** | 17 | 18 | | | 8 | 27 | | |
| **Age, years (mean[SD])** | 56.94 (10.31) | 52.06 (16.55) | 1.028 (0.975-1.083) | 0.304 | 62.38 (6.89) | 52.07 (14.65) | 1.094 (0.991-1.209) | 0.075 |
| **Gender (male), n (%)** | 13 (76.5%) | 9 (50.0%) | 3.250 (0.761-13.889) | 0.112 | 7 (87.5%) | 15 (55.6%) | 5.600 (0.603-52.004) | 0.130 |
| **DM duration, years (median[IQR])** | 8.00 (3.50-10.21) | 8.67 (2.44-16.00) | 0.978 (0.904-1.059) | 0.588 | 9.00 (3.25-10.27) | 7.33 (3.00-16.00) | 1.003 (0.914-1.100) | 0.957 |
| **DM treatment, n(%)** | | | | | | | | |
| *No drug therapy* | 1 (5.9%) | 0 (0.0%) | NA | 0.486 | 0 (0.0%) | 1 (3.7%) | NA | 1.000 |
| *Oral hypoglycaemic agents* | 6 (35.3%) | 6 (33.3%) | 1.091 (0.270-4.408) | 0.903 | 2 (25.0%) | 10 (37.0%) | 0.567 (0.095-3.363) | 0.532 |
| *Insulin* | 3 (17.6%) | 3 (16.7%) | 1.071 (0.185-6.217) | 0.939 | 2 (25.0%) | 4 (14.8%) | 1.917 (0.281-13.080) | 0.507 |
| *Insulin and oral hypoglycaemic agents* | 7 (41.2%) | 9 (50.0%) | 0.700 (0.184-2.664) | 0.601 | 4 (50.0%) | 12 (44.4%) | 0.800 (0.165-3.885) | 0.782 |
| **Laboratory findings** | | | | | | | | |
| *FBG, mmol/L (mean[SD])* | 6.68 (1.25) | 7.35 (1.93) | 0.768 (0.498-1.182) | 0.230 | 6.71 (1.31) | 7.12 (1.74) | 0.855 (0.519-1.409) | 0.539 |
| *2h-PBG, mmol/L (mean[SD])* | 11.27 (2.55) | 10.46 (2.44) | 1.148 (0.868-1.518) | 0.332 | 11.34 (2.58) | 10.71 (2.50) | 1.106 (0.809-1.513) | 0.527 |
| *HbA1c, % (median[IQR])* | 7.70 (6.00-8.65) | 7.50 (6.25-8.80) | 1.185 (0.791-1.775) | 0.411 | 7.30 (6.43-8.10) | 7.50 (6.18-9.10) | 0.845 (0.511-1.396) | 0.510 |
| *SCr, umol/L (median[IQR])* | 70.00 (57.00-78.00) | 64.50 (55.25-72.25) | 1.022 (0.985-1.061) | 0.245 | 73.50 (63.25-105.75) | 64.00 (53.00-72.00) | 1.049 (1.001-1.098) | 0.044[a] |
| *BUN, mg/L (mean[SD])* | 5.81 (2.23) | 5.16 (1.62) | 1.204 (0.833-1.741) | 0.322 | 6.96 (2.25) | 5.04 (1.64) | 1.775 (1.051-2.998) | 0.032[a] |
| *UMAlb, mg/L (median[IQR])* | 6.85 (2.45-32.15) | 3.00 (1.23-7.93) | 1.078 (0.978-1.189) | 0.130 | 8.80 (3.20-53.00) | 3.60 (1.60-13.90) | 1.001 (0.986-1.016) | 0.939 |
| *UCr, mmol/L (mean[SD])* | 10.14 (5.24) | 7.71 (3.86) | 1.133 (0.945-1.359) | 0.179 | 10.84 (4.93) | 8.50 (4.63) | 1.108 (0.906-1.355) | 0.316 |
| *ACR, mg/g Cr (median[IQR])* | 6.00 (3.00-26.00) | 3.00 (1.00-7.75) | 1.004 (0.993-1.016) | 0.452 | 7.00 (4.00-31.00) | 3.00 (2.00-20.00) | 1.004 (0.985-1.022) | 0.696 |
| *24hUP, g/24h (median[IQR])* | 0.13 (0.08-0.21) | 0.13 (0.08-0.18) | 25.960 (0.000-8.039×$10^6$) | 0.614 | 0.10 (0.08-0.21) | 0.14 (0.09-0.19) | 0.071 (0.000-308.814) | 0.536 |

[a] $P < 0.05$

**Abbreviations:** 2h-PBG: 2-hour postprandial blood glucose; 24hUP: 24 hours urine protein; ACR: albumin creatinine ratio; BUN: blood urea nitrogen; DM: diabetic mellitus; DM-NoDR: diabetes mellitus without clinically visible diabetic retinopathy; FBG: fasting blood glucose; IQR: interquartile range; NA: not applicable; NPA: nonperfusion area; OR: odds ratio; SCr: serum creatinine; SD: standard deviation; UCr: urine creatinine; UMAlb: urine microalbumin.

## 1.3 Supplementary Table 3. Univariable logistic regression analysis of potential related factors in DM-NoDR eyes with and without retinal microvascular changes in the peripheral area.

| | NPA | No-NPA | OR | P | Capillary tortuosity | No-Capillary tortuosity | OR | P |
|---|---|---|---|---|---|---|---|---|
| **Number, n** | 29 | 6 | | | 22 | 13 | | |
| **Age, years (mean[SD])** | 54.86 (14.09) | 52.33 (13.98) | 1.013 (0.953-1.076) | 0.682 | 55.86 (12.60) | 52.00 (16.10) | 1.021 (0.971-1.073) | 0.427 |
| **Gender (male), n (%)** | 18 (62.1%) | 4 (66.7%) | 0.818 (0.128-5.233) | 0.832 | 15 (68.2%) | 7 (53.8%) | 1.837 (0.448-7.539) | 0.399 |
| **DM duration, years (median[IQR])** | 10.00 (2.00-16.00) | 6.17 (3.75-12.25) | 1.040 (0.924-1.172) | 0.514 | 10.00 (0.94-17.75) | 7.00 (4.00-10.50) | 1.052 (0.960-1.152) | 0.276 |
| **DM treatment, n(%)** | | | | | | | | |
| *No drug therapy* | 1 (3.4%) | 0 (0.0%) | NA | 1.000 | 1 (4.5%) | 0 (0.0%) | NA | 1.000 |
| *Oral hypoglycaemic agents* | 9 (31.0%) | 3 (50.0%) | 0.450 (0.076-2.677) | 0.380 | 7 (31.8%) | 5 (38.5%) | 0.747 (0.178-3.129) | 0.689 |
| *Insulin* | 6 (20.7%) | 0 (0.0%) | NA | 0.561 | 3 (13.6%) | 3 (23.1%) | 0.526 (0.089-3.103) | 0.478 |
| *Insulin and oral hypoglycaemic agents* | 13 (44.8%) | 3 (50.0%) | 0.813 (0.140-4.721) | 0.817 | 11 (50.0%) | 5 (38.5%) | 1.600 (0.398-6.458) | 0.509 |
| **Laboratory findings** | | | | | | | | |
| *FBG, mmol/L (mean[SD])* | 6.90 (1.72) | 7.60 (1.17) | 0.767 (0.441-1.333) | 0.347 | 6.76 (1.78) | 7.47 (1.34) | 0.758 (0.486-1.182) | 0.221 |
| *2h-PBG, mmol/L (mean[SD])* | 10.98 (2.44) | 10.22 (2.90) | 1.149 (0.774-1.706) | 0.491 | 11.26 (2.66) | 10.17 (2.09) | 1.216 (0.891-1.660) | 0.217 |
| *HbA1c, % (median[IQR])* | 7.60 (6.30-8.50) | 7.45 (6.00-9.48) | 1.008 (0.604-1.682) | 0.977 | 7.50 (6.30-8.35) | 7.50 (6.15-10.20) | 0.746 (0.489-1.138) | 0.174 |
| *SCr, umol/L (median[IQR])* | 68.00 (57.00-74.00) | 61.50 (43.75-75.75) | 1.020 (0.969-1.073) | 0.455 | 68.50 (57.00-75.25) | 62.00 (49.50-73.00) | 1.010 (0.974-1.047) | 0.606 |
| *BUN, mg/L (mean[SD])* | 5.39 (2.02) | 5.90 (1.54) | 0.876 (0.564-1.360) | 0.555 | 5.39 (1.71) | 5.63 (2.34) | 0.935 (0.654-1.336) | 0.711 |
| *UMAlb, mg/L (median[IQR])* | 3.75 (1.90-15.40) | 4.80 (2.15-16.23) | 0.982 (0.902-1.067) | 0.663 | 4.25 (2.20-16.65) | 4.15 (0.93-16.93) | 1.007 (0.992-1.022) | 0.383 |
| *UCr, mmol/L (mean[SD])* | 8.96 (4.37) | 8.66 (7.14) | 1.015 (0.802-1.283) | 0.902 | 10.04 (4.38) | 6.90 (4.74) | 1.204 (0.963-1.505) | 0.103 |
| *ACR, mg/g Cr (median[IQR])* | 4.00 (2.00-20.00) | 3.00 (3.00-124.50) | 1.001 (0.992-1.010) | 0.839 | 4.50 (2.00-20.00) | 3.00 (1.75-59.25) | 1.013 (0.986-1.039) | 0.351 |
| *24hUP, g/24h (median[IQR])* | 0.13 (0.08-0.21) | NA | NA | NA | 0.10 (0.08-0.15) | 0.21 (0.18-0.74) | 0.000 (0.000-4.220×$10^5$) | 0.214 |

**Abbreviations:** 2h-PBG: 2-hour postprandial blood glucose; 24hUP: 24 hours urine protein; ACR: albumin creatinine ratio; BUN: blood urea nitrogen; DM: diabetic mellitus; DM-NoDR: diabetes mellitus without clinically visible diabetic retinopathy; FBG: fasting blood glucose; IQR: interquartile range; NA: not applicable; NPA: nonperfusion area; OR: odds ratio; SCr: serum creatinine; SD: standard deviation; UCr: urine creatinine; UMAlb: urine microalbumin.
